# Faecal DNA Metabarcoding for Diet Analysis of Endangered Fish Species, *Odontobutis obscurus*

**DOI:** 10.3390/ani14213083

**Published:** 2024-10-25

**Authors:** Kanghui Kim, Kyung-A You, Jeong-Hui Kim, Sang-Hyeon Park, Seung-Ho Baek, Kwang-Seuk Jeong, Gea-Jae Joo, Hyunbin Jo

**Affiliations:** 1 Department of Pet Health Care, Busan Health University, Busan 49318, Republic of Korea; kgh780@bhug.ac.kr; 2 Water Environment Research Department, National Institute of Environmental Research, Incheon 22689, Republic of Korea; angelka@korea.kr; 3 EcoResearch Incorporated, Gongju 32588, Republic of Korea; kjh@ecoresearch.co.kr (J.-H.K.); psh@ecoresearch.co.kr (S.-H.P.); bsh@ecoresearch.co.kr (S.-H.B.); 4 Department of Nursing Science, Busan Health University, Busan 49318, Republic of Korea; kjeong@bhug.ac.kr; 5 Department of Integrated Biological Science, Pusan National University, Busan 46241, Republic of Korea; gjjoo@pusan.ac.kr

**Keywords:** dark sleeper, endangered species, conservation translocation, next-generation sequencing

## Abstract

Understanding the ecological characteristics of endangered species is essential for their management and conservation. The dark sleeper (*Odontobutis obscurus*) is a carnivorous freshwater fish that has been designated an endangered species in South Korea because of its highly restricted habitat range and small population size. However, ecological research on prey items for this species remains severely limited globally, posing challenges for establishing conservation measures such as translocation management. In this study, the food sources and feeding strategies of *O. obscurus* were identified through non-invasive faecal DNA metabarcoding. A total of 24 *O. obscurus* specimens were collected from Sanyang Stream on Geoje Island, and faecal DNA was amplified using mitochondrial cytochrome c oxidase I (COI) primer. As a result, 13 prey items were identified, comprising fish and benthic invertebrates. Dietary metrics revealed the overall food composition and major food source of *O. obscurus*, and the Costello method enabled graphical diet analysis, suggesting that *O. obscurus* is a generalist, feeding on a wide variety of food sources. The food selectivity index suggested that *O. obscurus* prefers specific prey items, such as genus *Misgurnus*, *Zacco* (fish), *Radix* (gastropod), and *Ephemera* (mayfly). This molecular method facilitated the diet analysis of an endangered fish species that cannot undergo dissection, providing crucial information for their management, particularly regarding translocation efforts.

## Simple Summary

For the management and conservation of endangered species, we must understand their ecological characteristics. In this study, we extracted DNA from the faeces of *Odontobutis obscurus*, an endangered fish species in South Korea, and identified their food sources and feeding strategies without sacrificing the individuals. We detected 13 prey items and suggested that *O. obscurus* feeds on a wide variety of food sources and prefers specific prey items. This molecular method facilitated the diet analysis of an endangered fish species that cannot undergo dissection, providing crucial information for their management, particularly regarding translocation efforts.

## 1. Introduction

The dark sleeper, *Odontobutis obscurus* (Temminck and Schlegel, 1845) is a carnivorous freshwater fish native to East Asia [1]. As top predators, they play an important role in maintaining the structure and function of the ecosystem [1,2], and they also have biogeographic value for studying the past geographical connections of the East Asian region [3]. However, in South Korea, they are critically endangered because of their highly restricted habitat range and small population [4] and have been designated as Endangered Wildlife Class I by the Ministry of Environment. They were first reported in South Korea by Chae [5], exclusively inhabiting the Sanyang Stream watershed on Geoje Island [6], where they form an isolated population. Moreover, river maintenance work to prevent flooding in Sanyang Stream area (e.g., embankment construction) can affect the overall physical environment of the stream, such as river width, water depth, flow velocity, and substrate [7]. These changes could threaten their sole habitat, necessitating urgent translocation to alternative habitats.

Translocation is the process of establishing populations of species outside their natural range, providing new opportunities for saving endangered species [8,9]. However, the success of translocation depends on many factors (e.g., predation, behaviour, genetic diversity, invasion, disease, etc.) and can have unexpected impacts on the species and their new habitats [8,9,10,11,12]. Therefore, risk assessments, with baseline knowledge on the ecological characteristics of the species and thorough investigation of the new habitat, should precede any translocation efforts [8,12,13]. Recently, for example, pre-release surveys using radio telemetry tags, global positioning system (GPS) tags, camera traps, and environmental DNA have provided this basic information [14,15,16,17].

Nonetheless, ecological research on the food sources of *O. obscurus* remains severely limited worldwide. Zhu, et al. [18] reported that they are carnivorous and feed mainly on shrimps and small fish, but there were no related studies in South Korea, where they are protected as endangered species and cannot be sacrificed in experiments. Since diet analysis is the main key for comprehending predator–prey interactions and food web dynamics [19,20], and artificial introduction of carnivores may have a significant impact on the population of prey species and balance of the ecosystem [21,22], a non-invasive method to thoroughly understand the prey of *O. obscurus* in the Sanyang Stream is essential for successful translocation.

Faecal DNA metabarcoding enables non-invasive and high-resolution analysis of food sources without sacrificing individuals [23,24,25]. This method uses polymerase chain reactions (PCR) with universal or group-specific primers and next-generation sequencing (NGS) techniques, and researchers can obtain accurate and extensive information on prey items based on DNA sequences from faecal samples [26,27]. This molecular analysis allows fir prey identification at the species level based on DNA database, and furthermore, it is applicable to soft-bodied and small-sized prey [24]. It has been applied to a taxonomically wide range of animals, including endangered species [23,28,29,30], to successfully identify their prey targets.

In this study, we comprehensively identified the prey items and feeding strategies of the endangered *O. obscurus* through non-invasive faecal DNA metabarcoding. Field sampling was conducted in the Sanyang Stream watershed, and mitochondrial cytochrome c oxidase I (COI) primers were used to target all eukaryotic DNA. For this purpose, we used dietary metrics to indicate the overall food composition. In addition, we performed graphical diet analysis using the Costello method and examined prey preferences of *O. obscurus* using field survey data and food selectivity index. Our study facilitated the examination of food sources of an endangered fish species that cannot be dissected, providing crucial information for their management, particularly in the context of establishing alternative habitats and translocation efforts.

## 2. Materials and Methods

### 2.1. Ethical Approval

In accordance with the “Wildlife Protection and Management Act”, we obtained a permit for the capture and release of endangered species (No. 2018-31) from the Nakdong River Basin Environmental Office. According to the permit, the field survey was conducted without damage to the habitat, and captured individuals were immediately released into the field after sampling was completed.

### 2.2. Study Sites and Sample Collection

**Figure 1:**
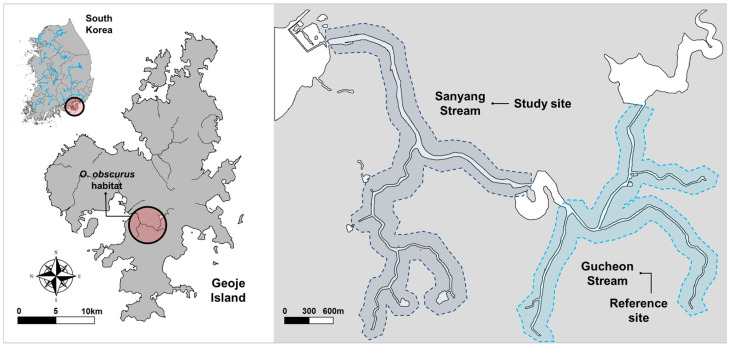
Map of the Sanyang Stream watershed, the habitat of *O. obscurus*. The Sanyang Stream watershed includes Sanyang Stream, Gucheon Stream, and their tributaries. Fish surveys including *O. obscurus* sampling were conducted in Sanyang Stream and its tributaries, and Gucheon Stream was a reference site for benthic invertebrate survey data.

In South Korea, *Odontobutis obscurus* (Temminck and Schlegel, 1845), previously called *O. obscura*, exclusively inhabits the Sanyang Stream watershed on Geoje Island (Figure 1). The island is located in the southern sea of the Korean Peninsula (34°51′29″ N 128°37′06″ E), with an annual temperature of 24 °C in the summer season and 3.9 °C in the winter season under a monsoonal influence. Annual precipitation is approximately 1900 mm, which is concentrated in the summer rainy season. The Sanyang Stream is a small river approximately 12.28 km long located in the western part of the island, and the watershed includes Sanyang and Gucheon Stream and their tributaries. It exhibits characteristics of complex substrate types and dense vegetation [5].

Fish surveys were conducted from January to September in 2019 (*n* = 5) in Sanyang Stream and its tributary, Buchun Stream. Cast nets (7 × 7 mm mesh size) and scoop nets (4 × 4 mm mesh size) were used, and collected fish species were identified according to Kim and Park [31] and organised according to Nelson, et al. [32]. We captured a total of 55 *O. obscurus* individuals with scoop nets and obtained faecal samples from 24 specimens collected between June and July. The total length (mm, from the tip of the snout to the tip of the tail), standard length (mm, from the tip of the snout to the posterior end of the last vertebra), and weight (g) of each specimen were measured, and they were placed in distilled water for a short time to prevent potential contamination. Faecal samples (*n* = 24) were obtained by gently squeezing the abdomen of the individuals and collected with tweezers. Disposable gloves and tweezers were used for each sample to minimise cross contamination. The samples were stored in 100% ethanol, transported to the laboratory for refrigerated storage, and stored at −80 °C until further analysis.

We also measured water quality and physical environmental factors at each capture point. Water temperature (°C), dissolved oxygen (mg/L), electrical conductivity (us/cm), salinity (psu), and pH were measured using YSI ProPlus (YSI, Yellow Springs, OH, USA). Substrate types were determined through scuba diving surveys below a depth of 1 m, according to Cummins [33]. Water depth and flow velocity were measured using a Swoffer Model 2100 Current Velocity Meter (Swoffer, Sumner, WA, USA).

### 2.3. DNA Metabarcoding

The entire DNA metabarcoding process was conducted in a sterile laboratory. Faecal samples were homogenised using sterilised homogeniser pestles. Each sample of 25 mg was subsampled using sterilised spoons, and genomic DNA was extracted using a DNeasy Blood and Tissue Kit (Qiagen, Hilden, Germany), with a final elution volume of 200 uL, according to the manufacturer’s instructions. The extracted DNA samples were stored at −20 °C.

Two consecutive PCR steps were performed for next-generation sequencing (NGS) processing. Negative and positive controls were employed in each PCR step. To prevent excessive amplification of self-DNA (i.e., host DNA of the predator itself), we customised several blocking primers sets (Table A1). However, they showed low efficiency in the pre-test, so we did not use the blocking primers in this study.

The first PCR was performed using mitochondrial cytochrome c oxidase I (COI) primer set, mlCOIintF and jgHCO2198 (Table 1), targeting universal eukaryotes [34]. The primers were tagged with Illumina adapters, and one or two PCR replicates per sample were performed until PCR succeed (i.e., the target band is clearly identified after gel electrophoresis without any extra bands). We used AccuPower HotStart PCR PreMix (Bioneer, Deajeon, Republic of Korea), and PCR reaction volume was 20 µL (1 µL DNA template, 1 µL forward primer (10 µM), 1 µL reverse primer (10 µM), and 17 µL distilled water). The PCR conditions consisted of 1 cycle of initial denaturation (95 °C, 5 min), 35 cycles of denaturation (95 °C, 30 s), annealing (55 °C, 30 s), and extension (72 °C, 1 min), and 1 cycle of final extension (72 °C, 7 min). After the first PCR, we checked the size of the products (approximately 345 bp) through 1.5% agarose gel electrophoresis. When the first PCR replicate failed, we extended the denaturation step to 40 cycles for the second PCR replicate. The purified first PCR products were stored at −20 °C.

The subsequent process was performed through the NGS service of THERAGEN BIO Co. (Seongnam-si, Gyeonggi-do, Republic of Korea). The second PCR was performed using KAPA HiFi HotStart ReadyMix (KAPA Biosystems, Wilmington, MA, USA) and Nextera XT Index Kit v2 (Illumina, San Diego, CA, USA). The volume of the PCR reaction solution was 25 µL (2.5 µL first PCR product, 2.5 µL forward index, 2.5 µL reverse index, 12.5 µL KAPA mix, 5 µL distilled water). The PCR conditions consisted of 1 cycle of initial denaturation (95 °C, 3 min), 10 cycles of denaturation (95 °C, 30 s), annealing (55 °C, 30 s), and extension (72 °C, 30 s), and 1 cycle of final extension (72 °C, 5 min). Subsequently, we checked the size of the products through 1.5% agarose gel electrophoresis and stored them at −20 °C.

The PCR products were purified via a bead clean-up process using 28 µL of AMPure XP beads (Beckman Coulter, Indianapolis, IN, USA) per sample. The samples were then quantified using a DeNovix QFX Fluorometer with a DeNovix dsDNA Ultra High Sensitivity Assay (DeNovix, Wilmington, DE, USA) and pooled equimolarly to produce a single library, according to the manufacturer’s protocol. The generated library was stored at −20 °C and sequenced on an Illumina MiSeq platform (2 × 250 paired-end; Illumina, San Diego, CA, USA).

### 2.4. Bioinformatics

Sequencing data were processed using QIIME 2 [35]. The demultiplexed raw sequences (FASTQ files) were merged into a single sequence and clustered into OTUs at a 97% OTU cutoff value. We used relative copy number threshold (≥0.01% of total number of reads) to remove low-level background noise such as secondary predation (e.g., prey of the prey), contamination, and sequencing errors [36,37]. Subsequently, the remaining OTUs were searched in the National Center for Biotechnology Information (NCBI) database (Release 256.0; June 2023) using BLASTN. The identity percent threshold (≥97%) was used, and the OTUs searched with *O. obscurus* or “no match” were excluded. Additionally, the OTUs that were not considered prey items for *O. obscurus* (e.g., fungi, Chromista, and cow) were removed. We finally assigned taxa by adjusting the identification level through comparison with the field survey data (Table S1). The obtained sequences were deposited in the NCBI repository under the accession number PRJNA1044277.

### 2.5. Dietary Analyses

#### 2.5.1. Dietary Metrics

The metabarcoding data were presented using two dietary metrics, frequency of occurrence and relative read abundance [37] to indicate overall prey composition detected in faecal samples of *O. obscurus*. The frequency of occurrence (FOO) is the number of samples containing a given prey item. A high FOO value suggests that the prey item is widespread and common in the study area. The relative read abundance (RRA) represents the average of the relative number of reads a prey item occupies in each sample. A high RRA indicates that the prey item is abundant and is represented by a large number of reads in the samples. %FOO and %RRA for prey item *i* were calculated as follows:

$${\%FOO}_{i}=\frac{1}{S}\sum_{k=1}^{S}I_{i,k}\times 100\%,$$

$${\%RRA}_{i}=\frac{1}{S}\sum_{k=1}^{S}\frac{n_{i,k}}{\sum_{i=1}^{T}n_{i,k}}\times 100\%,$$

where *T* is the number of prey items, *S* is the number of samples, *I* is an indicator function such that *I_i,k_* = 1 if prey item *i* is present in sample *k*, and 0 otherwise, and *n_i,k_* is the number of reads of prey item *i* in sample *k*.

#### 2.5.2. Costello Method

**Figure 2:**
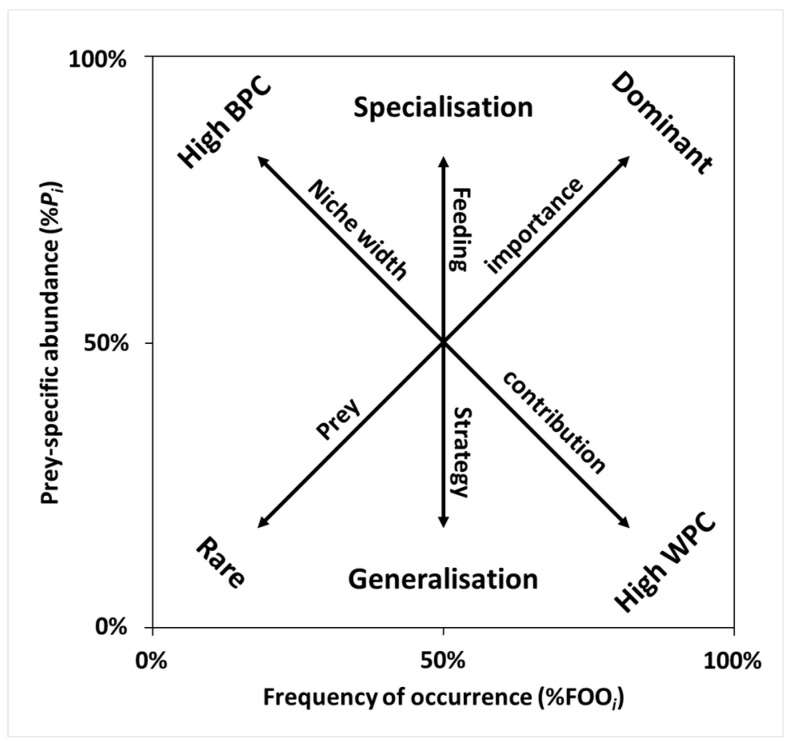
Theoretical diagram of the Costello method. The distribution of the points (each prey item) along the axes provides a graphical interpretation of the feeding strategy, prey importance, and niche width contribution (BPC: between-phenotype component; WPC: within-phenotype component).

To examine the dietary characteristics of *O. obscurus* graphically, we employed the Costello method [38]. The prey-specific abundance (%*P_i_*) was plotted against %FOO on a two-dimensional graph (Figure 2). To apply this method to the sequencing data, %*P_i_* for prey item *i* was calculated as follows:

$$\%P_{i}=\sum S_{i}/\sum S_{t_{i}}\times 100\%,$$

where $S_{i}$ is the sum of reads for prey item *i*, and $S_{t_{i}}$ is the sum of reads for samples including prey item *i*.

The vertical axis represents a feeding strategy in terms of specialisation (i.e., predation concentrated on several prey items) and generalisation (i.e., occasional predation for more diverse prey items). The diagonal axis from the upper left to the lower right represents the niche width contribution in terms of the high between-phenotype component (BPC; use of different resources between the individuals) and the high within-phenotype component (WPC; use of various resources within an individual). The diagonal axis from the upper right to the lower left represents the importance of the prey in terms of dominance and rarity.

#### 2.5.3. Selectivity Index and Reference Data

To examine the prey selection of *O. obscurus* for the available food in the habitat, we used Jacobs’s selectivity index [39]. Preferences for fish and benthic invertebrates were calculated separately, based on the %RRA of the prey items and field survey data. We used our fish survey data (at the same site as *O. obscurus* sampling points) and referred to benthic invertebrate survey data from the Water Environment Information System (in 2019, Gucheon stream, *n* = 2; Figure 1). Prey items were analysed at the genus level, except for the family Chironomidae, owing to the identification limit of the field survey. The Jacobs’s index D for food item *i* was calculated as follows:

$$D_{i}=\frac{r_{i}-p_{i}}{r_{i}+p_{i}-2r_{i}p_{i}}$$

where *r_i_* is the RRA of prey item *i*, and *p_i_* is the ratio of prey item *i* to the total number of fish or benthic invertebrates surveyed in the field. The index ranges from −1 (negative selection) to 1 (positive selection), and a value of 0 means no preference or avoidance.

#### 2.5.4. Statistical Analysis

Statistical analyses were performed using Paleontological Statistics (PAST) 4.05 (Natural History Museum, Oslo, Norway). Rarefaction analysis was performed to determine the sample size and the number of reads required to represent sufficient OTUs richness. Mann–Whitney U test [40] was used to compare the relative abundance and OTUs richness of the two major prey groups: fish and benthic invertebrates. Canonical correspondence analysis (CCA) [41] was used to investigate the correlation between environmental factors and prey items of *O. obscurus*, with %RRA data and normalised values of environmental factors. Subsequently, permutation tests for CCA were performed to determine the significance of the association.

## 3. Results

### 3.1. Overview of the Metabarcoding Results

**Figure A1:**
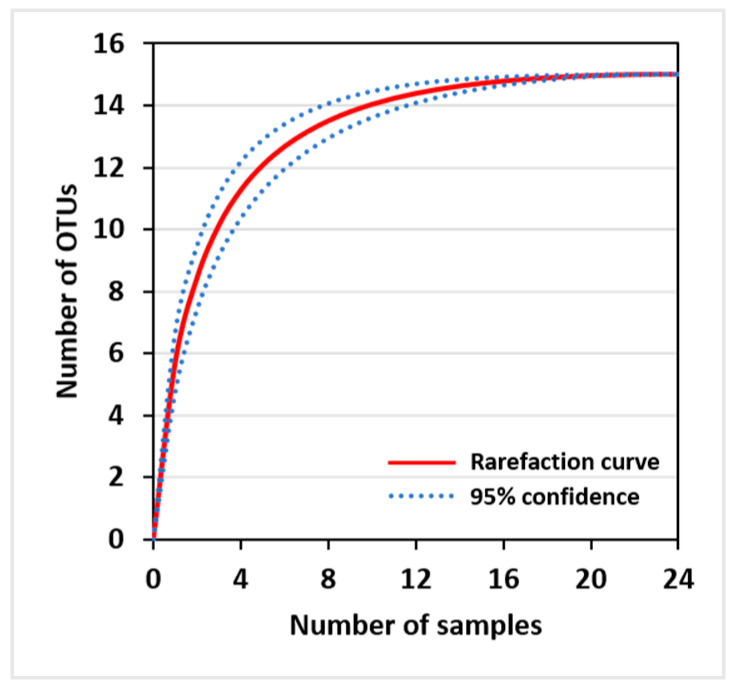
Rarefaction curves based on the number of OTUs and sample size.

DNA metabarcoding analysis produced 7,484,605 paired-end reads (average of 311,858 reads per sample) from 24 faecal samples, comprising 1020 unique OTUs. Through quality filtering with relative reads count threshold, 370 OTUs remained, which resulted in 15 taxa after taxonomic assignment (Table 2). *Zacco* sp. 2 and *Radix* sp. were initially identified as *Z. platypus* and *R. plicatula*, respectively; however, the species was evaluated as not living at the study site through a comparison with the field survey and was assigned at the genus level. The rarefaction curve indicates the saturation of the number of OTUs obtained via DNA metabarcoding (Figure A1).

### 3.2. Dietary Metrics and Comparison of the Two Major Prey Groups

**Figure 3:**
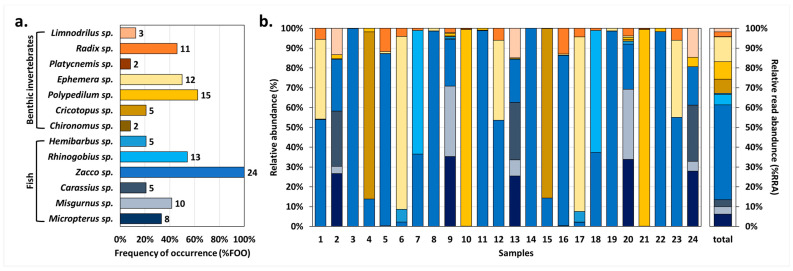
Overall prey items detected from the faecal samples of *O. obscurus* (*n* = 24). (**a**) Frequency of occurrence (%FOO) of each prey item. The number at the edge of the bars refers to the number of samples in which the prey item detected. (**b**) Relative read abundance (%RRA) of each prey item in 24 samples. The colour legend of each prey item is same as in (**a**).

The dietary metrics, FOO and RRA, represented the overall prey composition of *O. obscurus* faeces (Figure 3). *Zacco* sp. exhibited the highest values for both FOO and RRA, being present in all samples (100.0%, 24/24 samples), and displayed the most abundant DNA reads on average (47.9%). Second, *Polypedilum* sp. appeared in 62.5% (15/24) of the samples, accounting for 8.9% of the DNA reads. In addition, *Ephemera* sp. (50.0% FOO, 12.5% RRA), *Cricotopus* sp. (20.8% FOO, 7.1% RRA), *Micropterus* sp. (33.3% FOO, 6.2% RRA), and *Rhinogobius* sp. (54.2% FOO, 5.2% RRA) had RRA values greater than 5%.

**Figure 4:**
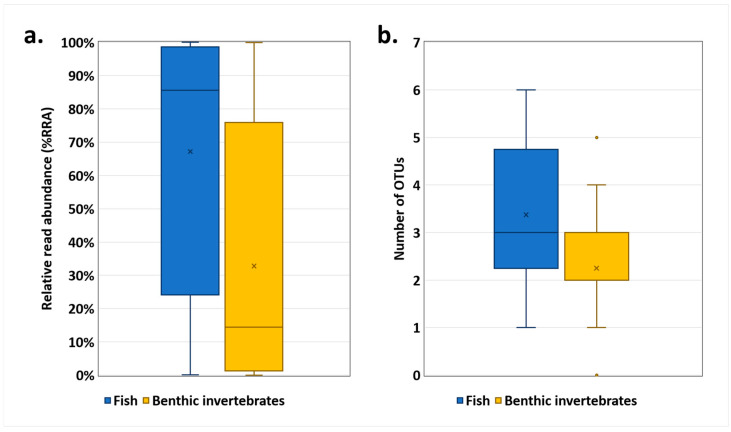
Box plot comparing (**a**) relative read abundance (%RRA) and (**b**) OTUs richness of two prey groups of *O. obscurus*: fish and benthic invertebrates. The fish group showed a significantly higher %RRA (Mann–Whitney U test: *p* = 0.0067) and OTUs richness (Mann–Whitney U test: *p* = 0.0083) than the benthic invertebrate group. The cross marks represent averages, the lines within the boxes are medians, and the dots outside the boxes represent outliers.

Moreover, in a comparison of the two major prey groups of *O. obscurus*, fish group displayed higher values than benthic invertebrates (Figure 4 and Figure S1). Based on a Mann–Whitney U test, the fish group showed significantly higher %RRA values than the benthic invertebrate group (*p* = 0.0067). In addition, the OTUs richness of the fish group was significantly higher than that of the benthic invertebrate group (*p* = 0.0083).

### 3.3. Graphical Diet Analysis Through Costello Method

**Figure 5:**
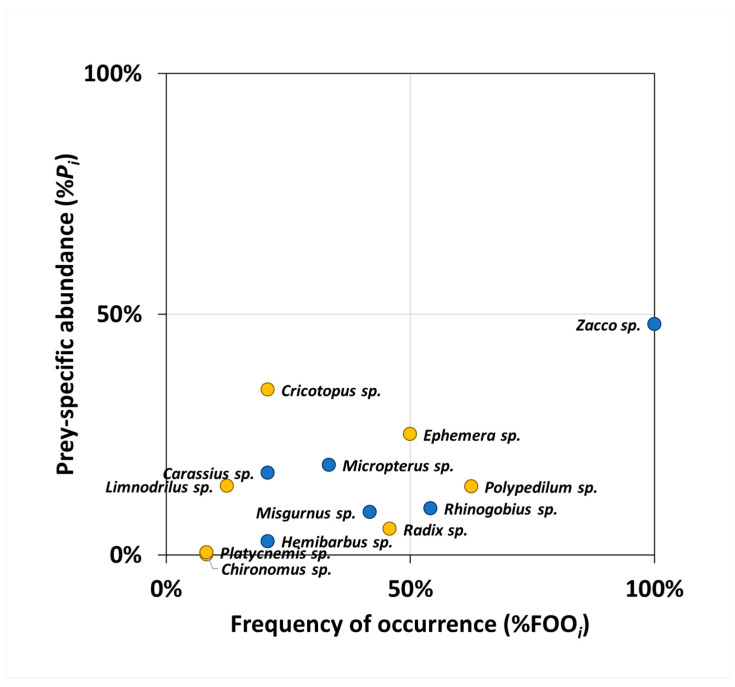
Diagram of the Costello method used to analyse the feeding habit of *O. obscurus*. The distribution of the points (each prey item detected in faecal samples—blue: fish group; yellow: benthic invertebrate group) provides a graphical interpretation of the feeding strategy, prey importance, and niche width contribution.

The diagram from the Costello method showed that all prey items detected in the faecal samples of *O. obscurus* were distributed in the lower part of the graph (Figure 5; %*P_i_* < 50%). *Zacco* sp., *Polypedilum* sp., and *Rhinogobius* sp. were positioned on the lower right (%FOO*_i_* > 50%, %*P_i_* < 50%), and most of the prey items were positioned on the lower left (%FOO*_i_* < 50%, %*P_i_* < 50%).

### 3.4. Comparison with Field Survey and Prey Selectivity of O. obscurus

**Figure 6:**
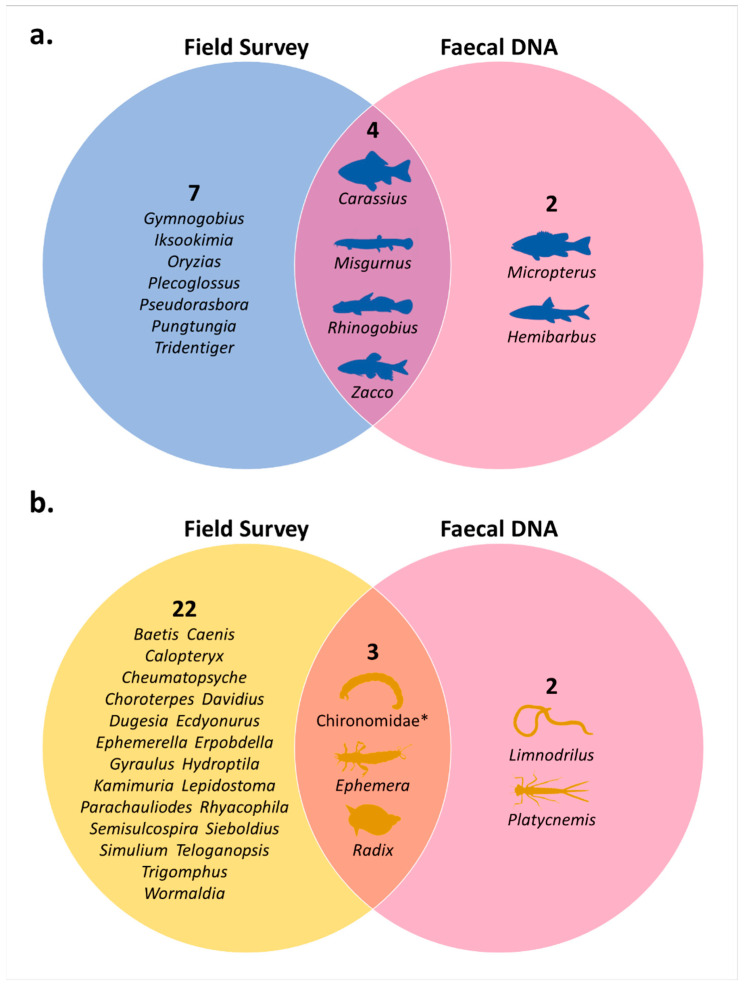
Venn diagrams illustrating (**a**) fish and (**b**) benthic invertebrates detected in field survey and faecal DNA metabarcoding. All taxa are presented at genus level except for the family Chironomidae marked with *.

In a comparison of the taxa detected in faecal DNA metabarcoding with field surveys at the genus level (except for the family Chironomidae), the prey items from faecal samples included only a portion of the taxa found in the field survey. In the fish group, of the 11 taxa identified in the field (survey at the same site as *O. obscurus* sampling points), only four were detected in the faecal DNA (Figure 6a). In the benthic invertebrate group, of the 25 taxa identified in the field (data from the reference site, Gucheon Stream; Figure 1), only 3 were detected in the faecal DNA (Figure 6b).

**Figure 7:**
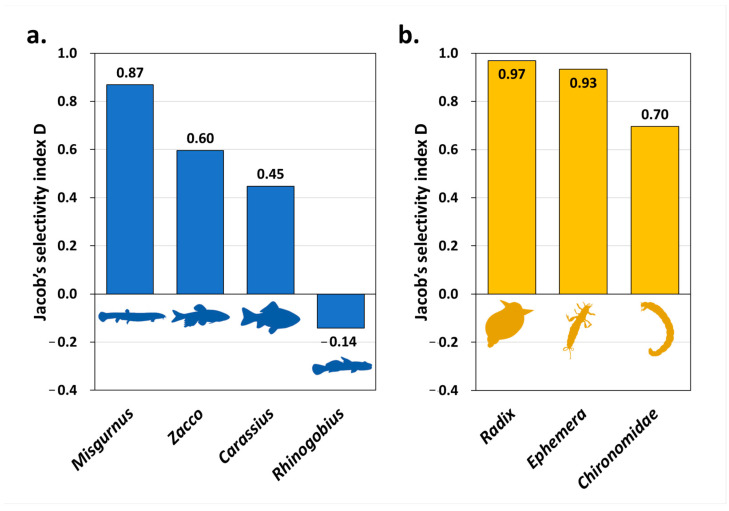
Prey selectivity of *O. obscurus* for fish (**a**) and benthic invertebrates (**b**) based on the relative read abundance (RRA) values of prey items detected in faecal samples and the population surveyed in the field. Prey items were analysed at the genus level except for the family Chironomidae. The index appears as a value between −1 (negative selection) and 1 (positive selection), and a value of 0 means no preference or avoidance.

Moreover, we calculated *O. obscurus*’ selectivity for prey items (Figure 7 and Figure S2) based on both faecal DNA metabarcoding and field survey data (Table S1). In the fish group, *O. obscurus* showed a preference for genus *Misgurnus*, *Zacco*, and *Carassius* and no preference or avoidance for genus *Rhinogobius*. In the benthic invertebrate group, *O. obscurus* preferred genus *Radix* and *Ephemera* and family Chironomidae. The number of prey items detected only by faecal metabarcoding (D = 1) or the field survey (D = −1) was 4 (2 fish and 2 benthic invertebrates) and 28 (7 fish and 21 benthic invertebrates), respectively (Figure S2).

Additionally, we performed CCA to investigate the correlation between environmental factors (Table 3) and prey items of *O. obscurus* (Figure S3), but permutation tests for CCA showed that the association was not statistically significant (*p* value for each axis > 0.6).

## 4. Discussion

In the present study, we non-invasively identified prey items of *O. obscurus* using faecal eDNA metabarcoding and applied conventional analytical methods to the DNA data to find out their dietary characteristics. We successfully identified 13 prey items at genus level, including fish and benthic invertebrates, and computed dietary metrics and food selectivity from DNA reads counts.

### 4.1. Dietary Characteristics of O. obscurus and Suggestions for Translocation

*Odontobutis obscurus* in South Korea is critically endangered due to its small population and limited habitat and urgently requires conservation measures, including translocation management [4]. Knowledge of food web and dietary characteristics is essential for all conservation strategies [42,43,44], and a comprehensive understanding of food source composition, feeding strategies, and food selectivity is required for stable translocation of *O. obscurus*.

Our results suggest a list of prey items thought to be the main food source for *O. obscurus* in Sanyang Stream (Table 2, Figure 3). In particular, *Zacco* sp., which showed the highest value of dietary metrics (100.0% FOO, 47.9% RRA) and appeared to be the dominant species in the field surveys, was considered the most important prey item of *O. obscurus* in the Sanyang Stream. Also, our results suggest that fish, rather than benthic invertebrates, are the primary food source for *O. obscurus*. All detected fish OTUs showed a FOO of more than 20% (Figure 3), and their numbers of OTUs and RRA were significantly higher than those of benthic invertebrates (Figure 4), suggesting that fish is the general food source for *O. obscurus*. These results suggest that habitats with abundant fish food sources should be considered first for the successful translocation of *O. obscurus* and that places with higher densities of the genus *Zacco*, including *Z. koreanus*, *Z. platypus*, and *Z. temminckii*, would be suitable as alternative habitats.

Furthermore, we suggest that *O. obscurus* is a generalist with a wide niche breadth and high WPC, using a variety of resources within an individual (Figure 5). They particularly preferred certain prey items, such as *Misgurnus*, *Zacco*, *Radix*, and *Ephemera* (Figure 7), which indicated that rather than randomly feeding all the available resources, they exhibited some selectivity based on their predatory strategy or the ecological habits of each prey item. The finding that the prey items of *O. obscurus* were not significantly associated with environmental factors in their habitats also supports this prey selectivity (Figure S3). These results suggest that *O. obscurus*, unlike specialists who focus on one or a few prey items, can reliably adapt to alternative habitats based on a wide range of food sources [45]. Additionally, prey items such as *Misgurnus*, *Zacco*, *Radix*, and Chironomidae, which represented the positive selectivity index in this study, are common in freshwater areas [31,46], providing an advantage in establishing alternative habitats for *O. obscurus*’ translocation; whereas, with a high preference of 0.93, *Ephemera* spp., including *E. separigata*, *E. orientalis*, and *E. strigata*, mainly inhabited relatively clean streams or rivers [47,48], presenting considerations for the water quality of the alternative habitats.

In addition, we found that all 24 *O. obscurus* were captured in an environment with a gravelly substrate and flat water that was <60 cm deep (Table 3) [4,5]. These results are thought to be related to the ambush-type feeding behaviour of *O. obscurus*, which lies at the bottom and waits motionlessly for prey to approach before snatching it with its large mouth [2]. Therefore, similar to other ambush-type predators in the family Odontobutidae, a flat water with complex substrates and a low water depth would be suitable as new habitats.

In conclusion, our study on the diet of *O. obscurus* based on faecal DNA metabarcoding provides the following suggestions for the most suitable alternative habitats for translocation:

1. We suggest a habitat with a high density of fish and benthic invertebrates, particularly *Zacco* spp.
2. As a generalist, they have the potential to easily adapt to new alternative habitats. However, they showed preferences for certain prey items, so habitats covering such prey selectivity are more recommended.
3. We suggest clean flat water with complex substrates and a low water depth suitable for ambush-type predators.

These findings provide essential dietary information for managing *O. obscurus*. However, in order to implement translocation, many other variables and more in-depth questions still have to be considered [8,11,12]. In particular, because *O. obscurus* plays a key role in regulating the populations of other species as a top predator, the impact of their artificial introduction on the local ecosystem can be more significant and unpredictable [21,22]. Therefore, further research on *O. obscurus*, further environmental evaluation of the alternative habitats, further risk assessments, and further preparation of appropriate and effective plans should be thoroughly carried out.

### 4.2. Faecal DNA Metabarcoding for Diet Analysis of Endangered Fish Species

Faecal DNA metabarcoding is a non-invasive, accurate, and efficient tool for diet analysis, and many studies have used this molecular analysis approach to identify food sources for various animals [25,49]. In particular, mammals have been the major subject of faecal DNA metabarcoding because it is easy to obtain large amounts of faecal samples from them [49]. There have only been a few cases of this method being employed for fish due to the difficulty in collecting the faecal samples; therefore, many studies used stomach or gut contents attained through the dissection process rather than analysing faeces [50,51,52,53]. However, in this study, we identified prey items of an endangered fish species through faecal DNA metabarcoding and further revealed their dietary characteristics using conventional analytic methods such as the Costello method and the food selectivity index. This approach could be applicable to other endangered fish that cannot be sacrificed for dietary study and could be the most appropriate tool with which to provide critical dietary information for their management and conservation.

### 4.3. Limitations and Further Study

In this study, we were able to provide some important information about the dietary characteristics of *O. obscurus*, but this clearly needs to be developed through further studies. First, even though we confirmed that more than 20 samples could detect a sufficient number of prey items through rarefaction analysis (Figure A1), we were able to collect only a limited number of samples (*n* = 24) during the short-term surveys to preserve their population. In future studies, the most important objective will be to discover seasonal variations in the food sources of *O. obscurus* and to establish general data on their dietary characteristics with larger sample sizes. Second, we used the reference data to compare faecal DNA with field surveys (Figure 6, Table S1), but the study site and period are not exactly the same as our study. Because the difference in detected species may be exaggerated, this should be complemented in future studies. Additionally, we identified that some taxa not found in field surveys was detected in faecal DNA. It is possible that the taxa that was accidentally missing in the field survey was detected via high-resolution DNA metabarcoding, but the aforementioned limitations of the reference data still have to be considered. Lastly, further multifaceted studies, including diet breadth, niche overlap, temporal feeding patterns, and ontogenetic diet shifts, can improve the comprehensive understanding of *O. obscurus*’ feeding ecology and supplement our present results, helping to develop the most appropriate conservation and management strategies [19,20].

In addition, DNA metabarcoding for diet analysis has the potential risk of recovery biases [25,37]. For example, secondary predication, contamination, or technical biases may be the reason why DNA read counts cannot fully represent prey composition. In our study, this risk was minimised through quality filtering, including relative DNA read count thresholds. As such, DNA metabarcoding for diet analysis must manage these biases sufficiently.

Moreover, there are few analytical methods for dietary metabarcoding data, and most methods for classical visual inspection are difficult to apply. The Costello method used in this study is also a technique mainly used for classical diet analysis; when applied to diet metabarcoding data with higher resolution, the relative abundance may be dispersed in the detected prey items, resulting in a low overall value. Therefore, it is necessary to recognise and fully consider these biases when applying classic analytical methods to diet metabarcoding data and to overcome these methodological challenges by devising analytical techniques for dietary metabarcoding data and modifying existing techniques [37].

## 5. Conclusions

Our study identified the food sources and feeding strategies of *O. obscurus* through non-invasive faecal DNA metabarcoding. We detected 13 prey items from 24 faecal samples, and conventional dietary analyses suggested important information about the feeding strategy of *O. obscurus*. This molecular method facilitated the diet analysis of an endangered fish species that cannot undergo dissection, providing crucial information for their management, particularly regarding establishing alternative habitats and translocation efforts.

## Figures and Tables

**Table 1:** Table of primer information. The cytochrome oxidase I (COI) primer set, mlCOIintF and jgHCO2198, was used to target universal eukaryotes.

Target Organisms (Region)	Name	Sequences	References
Animal (COI)	mlCOIintF	GGWACWGGWTGAACWGTWTAYCCYCC	Leray et al. 2013 [34]
	jgHCO2198	TAIACYTCIGGRTGICCRAARAAYCA	

**Table 2:** List of prey items detected from the faecal samples of *O. obscurus* (*n* = 24) and BLASTn results of each prey item.

Prey Group	Class	Genus + Species	Max Score	Identity (%)	Query (%)	Genbank Accession
Fish	Actinopteri	*Micropterus salmoides*	579	100.0	100	MT455106.1
		*Misgurnus anguillicaudatus*	579	100.0	100	MF122502.1
		*Carassius cuvieri*	579	100.0	100	MT571744.1
		*Zacco* sp. 1	556	98.7	100	MT457508.1
		*Zacco* sp. 2	579	100.0	100	MT457518.1
		*Rhinogobius brunneus*	579	100.0	100	OL674307.1
		*Hemibarbus labeo*	573	99.7	100	OL674364.1
Benthic macro invertebrates	Insecta	*Chironomus flaviplumus*	534	97.4	100	MN521255.1
		*Cricotopus triannulatus*	579	100.0	100	LC050962.1
		*Polypedilum japonicum*	573	99.7	100	LC329191.1
		*Polypedilum yongsanensis*	579	100.0	100	NC_072650.1
		*Ephemera orientalis*	562	99.0	100	OL664518.1
		*Platycnemis phyllopoda*	573	99.7	100	KF257109.1
	Gastropoda	*Radix* sp.	564	99.0	100	LC658589.1
	Clitellata	*Limnodrilus* sp.	579	100.0	100	KY369698.1

**Table 3:** Table of *O. obscurus* sampling sites and date, environmental factor measurements, and size information of the collected *O. obscurus* samples.

Date	Sample Number	Water Quality					Physical Environment			Sample Length and Weight		
		Temp (°C)	DO (mg/L)	Conduc (us/cm)	Salinity (psu)	pH	Depth (cm)	Flow Velocity (m/s)	Substrate	TL (mm)	L (mm)	Weight (g)
4 June 2019	1	23.8	10.66	277.8	0.11	7.46	60	<0.1	gravel, boulder	86	72	7.8
	2	23.5	11.68	233.6	0.11	7.55	30	<0.1	gravel, sand	82	66	8.1
	3	24.0	11.74	277.0	0.11	7.71	30	<0.1	gravel, sand	96	81	13.8
	4	23.9	11.31	236.0	0.11	7.67	30	<0.1	gravel, sand	94	82	12.8
	5	23.7	9.83	202.2	0.11	7.57	20	<0.1	gravel, sand	82	67	6.8
	6	23.9	10.78	231.4	0.11	7.65	20	<0.1	gravel, sand	91	72	12.0
	7	24.2	10.04	230.5	0.11	7.62	40	<0.1	gravel, sand	89	71	9.9
	8	23.6	9.77	227.7	0.11	7.53	30	<0.1	gravel, sand	89	70	9.4
	9	23.5	9.80	225.1	0.11	7.51	40	<0.1	gravel, sand	90	76	10.5
	10	24.9	7.71	248.0	0.12	7.45	20	<0.1	gravel, sand, boulder	87	72	9.5
	11	25.7	7.73	254.8	0.12	7.46	10	<0.1	gravel, sand, boulder	98	81	12.1
	12	25.7	7.73	254.8	0.12	7.46	10	<0.1	gravel, sand, boulder	86	72	8.7
	13	24.6	9.25	255.1	0.12	7.42	20	<0.1	gravel, sand	96	82	11.8
	14	26.3	7.84	260.1	0.12	7.24	20	<0.1	gravel, sand, boulder	81	67	7.4
	15	26.3	7.84	260.1	0.12	7.24	20	<0.1	gravel, sand, boulder	83	65	9.2
	16	26.3	7.84	260.1	0.12	7.24	20	<0.1	gravel, sand, boulder	97	81	13.2
	17	25.5	9.07	249.0	0.11	7.18	20	<0.1	gravel, sand, boulder	88	75	9.8
	18	26.4	7.47	270.0	0.12	7.51	30	<0.1	gravel, sand, silt, boulder	84	67	7.6
	19	26.4	7.47	270.0	0.12	7.51	30	<0.1	gravel, sand, silt, boulder	83	66	8.4
	20	26.4	7.47	270.0	0.12	7.51	30	<0.1	gravel, sand, silt, boulder	84	71	7.1
	21	26.4	7.47	270.0	0.12	7.51	30	<0.1	gravel, sand, silt, boulder	83	69	8.7
17 July 2019	22	24.3	8.18	77.6	0.04	7.50	30	<0.1	gravel, sand	98	83	12.3
	23	24.3	8.18	77.6	0.04	7.50	30	<0.1	gravel, sand	83	68	8.5
	24	24.3	8.18	77.6	0.04	7.50	30	<0.1	boulder	98	80	12.2

Abbreviations: Temp, water temperature; DO, dissolved oxygen; Conduc, electrical conductivity; TL, total length; SL, standard length.

## Data Availability

Raw sequence reads are deposited to NCBI Nucleotide Database under the accession number PRJNA1044277.

## Supplementary Materials

The following supporting information can be downloaded at https://www.mdpi.com/article/10.3390/ani14213083/s1: Table S1: Field survey results; Figure S1: Bar graphs show (a) the relative read abundance (%RRA) and (b) the OTUs richness of the two prey groups, fish and benthic invertebrates, for each sample; Figure S2: The total prey preference of *O. obscurus* was based on the relative read abundance (RRA) values of prey items detected from faecal samples and populations surveyed in the field; Figure S3: Pairwise Pearson correlation of between normalised environmental factor measurements and RRA data of detected prey items.

## Appendix A

**Table A1:** This is Table of blocking primer information. Several customised blocking primers sets were developed but not applied in this study.

	Sequence (5′->3′)	Template strand	Length	Start	Stop	Tm	GC%	Length (bp)
Block_1_F	CCCCCGATATAGCCTTCCCT	Plus	20	179	198	60.25	60	143
Block_1_R	CAGGTTTCCTGCTAACGGGG	Minus	20	321	302	60.68	60	
