# Supplementary material for: Faecal DNA Metabarcoding for Diet Analysis of Endangered Fish Species, Odontobutis obscurus
Source: Animals (Basel). 2024 Oct 25;14(21):3083. doi: 10.3390/ani14213083 (PMC11545592; doi:10.3390/ani14213083)
Supplement: Supplementary file 1 [file animals-14-03083-s001.zip › animals-3251564-supplementary.pdf]

Faecal DNA Metabarcoding for Diet Analysis of Endangered Fish Species, *Odontobutis obscurus*

Kanghui Kim[1], Kyung-A You[2], Jeong-Hui Kim[3], Sang-Hyeon Park[3], Seung-Ho Baek[3], Kwang-Seuk Jeong[4], Gea-Jae Joo[5], Hyunbin Jo[1,*]

Supplementary data

Table S1. Field survey results.

Figure S1. Bar graphs show (**a**) the relative read abundance (%RRA) and (**b**) the OTUs richness of the two prey groups, fish and benthic invertebrates, for each sample.

Figure S2. The total prey preference of *O. obscurus* was based on the relative read abundance (RRA) values of prey items detected from faecal samples and populations surveyed in the field. Prey items were analysed at the genus level, except for the family Chironomidae. The index has a value between -1 (negative selection; no detection in eDNA metabarcoding) and 1 (positive selection; no detection in field surveys), and a value of 0 indicates no preference or avoidance.

Figure S3. Canonical correspondence analysis (CCA) plots displaying the correspondence of environmental factors with relative read abundance (%RRA) of prey items detected in the faecal samples of *O. obscurus*. Cross marks represent each sample (n = 24), and dots represent prey items (blue: fish, yellow: benthic invertebrates). Environmental factors, including water temperature (Temp), dissolved oxygen (DO), electrical conductivity (Conduc), salinity (Sal), pH and water depth (Depth), are displayed as green arrows. Permutation tests for CCA showed that the association was not statistically significant (p value for each axis > 0.6)

Table S1. Field survey results.

Taxonomic group	Taxa	Surveyed number
Fish	*Carassius auratus*	27
	Gymnogobius urotaenia	56
	Iksookimia longicorpa	111
	Misgurnus anguillicaudatus	4
	Oryzias latipes	196
	Plecoglossus altivelis	22
	Pseudorasbora parva	58
	Pungtungia herzi	6
	Rhinogobius brunneus	70
	Tridentiger brevispinis	9
	Zacco koreanus	258
	Zacco temminckii	5
Benthic invertebrates	*Baetis fuscatus*	12
	Baetis pseudothermicus	8
	Caenis KUa	19
	Calopteryx japonica	4
	Chironomidae spp. (non-red type)	64
	Choroterpes (Euthraulus) altioculus	123
	Davidius lunatus	4
	Dugesia sp.	8
	Ecdyonurus kibunensis	42
	Ecdyonurus levis	327
	Ephemera strigata	12
	Ephemerella kozhovi	19
	Gyraulus convexiusculus	4
	Hydroptila KUa	4
	Kamimuria coreana	4
	Lepidostoma KUa	8
	Parachauliodes asahinai	4
	Radix auricularia	4
	Rhyacophila nigrocephala	15
	Semisulcospira libertina	78
	Sieboldius albardae	8
	Simulium sp.	8
	Teloganopsis punctisetae	4
	Trigomphus citimus	4
	Wormaldia KUa	4
	Cheumatopsyche brevilineata	4
	Erpobdella lineata	8
	Semisulcospira gottschei	8

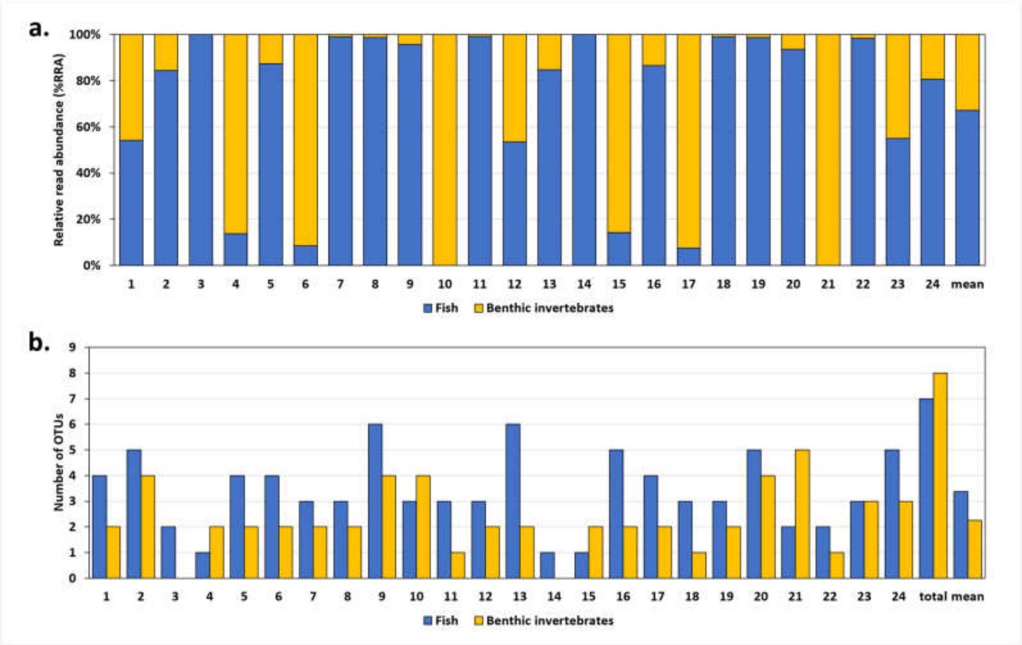

Figure S1. Bar graphs show (**a**) the relative read abundance (%RRA) and (**b**) the OTUs richness of the two prey groups, fish and benthic invertebrates, for each sample.

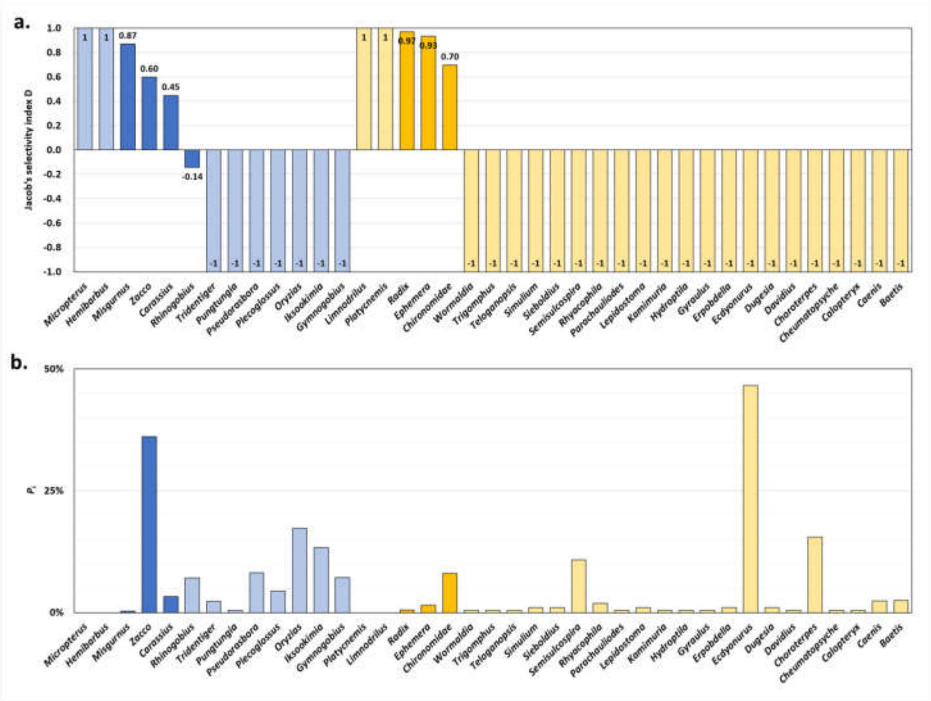

Figure S2. The total prey preference of *O. obscurus* based on the relative read abundance (RRA) values of prey items detected from faecal samples and populations surveyed in the field. Prey items were analysed at the genus level, except for the family Chironomidae. The index has a value between -1 (negative selection; no detection in eDNA metabarcoding) and 1 (positive selection; no detection in field surveys), and a value of 0 indicates no preference or avoidance.

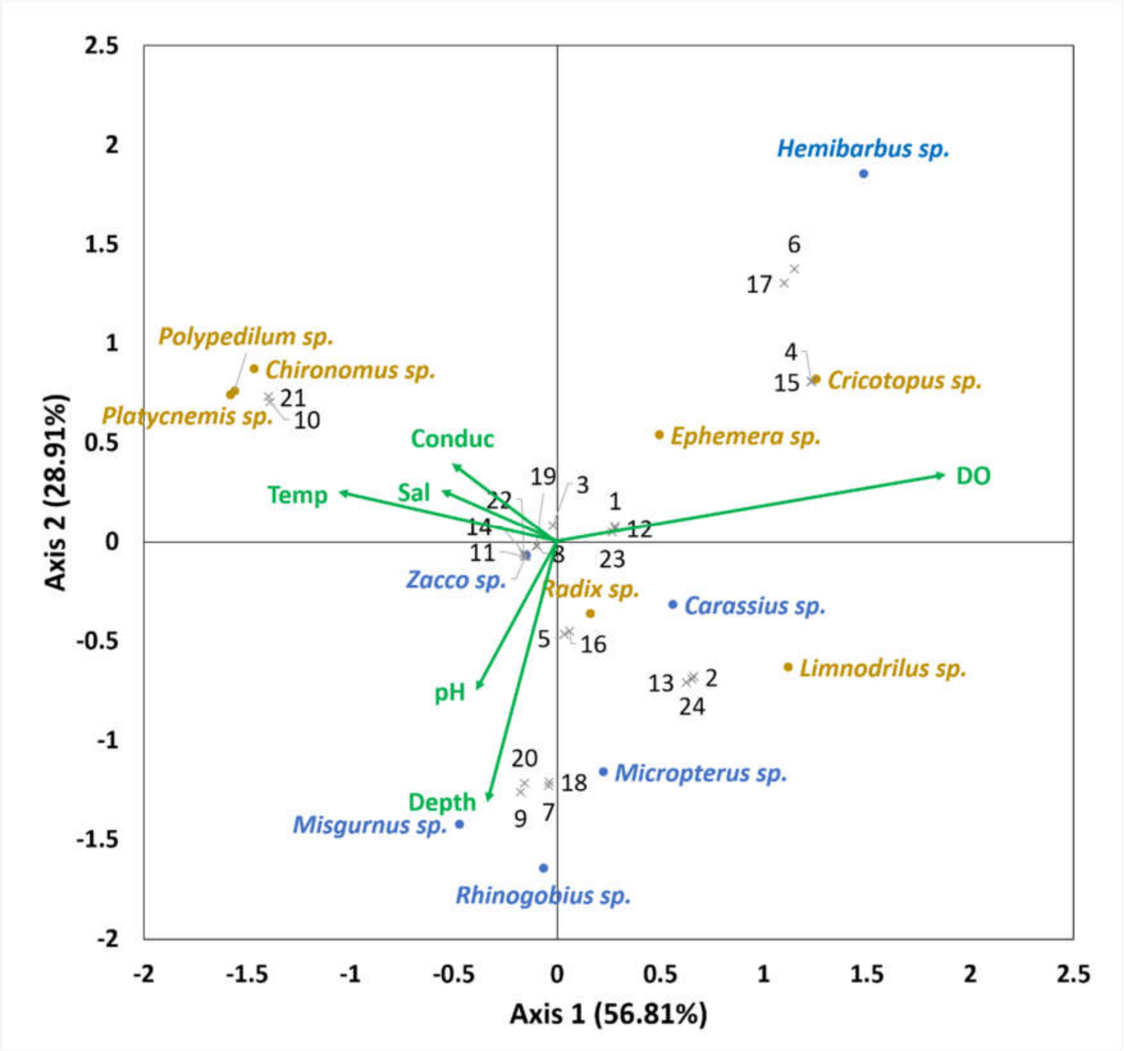

Figure S3. Canonical correspondence analysis (CCA) plots displaying the correspondence of environmental factors with relative read abundance (%RRA) of prey items detected in the faecal samples of *O. obscurus*. Cross marks represent each sample (*n* = 24), and dots represent prey items (blue: fish, yellow: benthic invertebrates). Environmental factors, including water temperature (Temp), dissolved oxygen (DO), electrical conductivity (Conduc), salinity (Sal), pH and water depth (Depth), are displayed as green arrows. Permutation tests for CCA showed that the association was not statistically significant (p value for each axis > 0.6)
